# Mercury in marine fish, mammals, seabirds, and human hair in the coastal zone of the southern Baltic

**DOI:** 10.1007/s11270-015-2735-5

**Published:** 2016-01-16

**Authors:** Magdalena Bełdowska, Lucyna Falkowska

**Affiliations:** Institute of Oceanography, University of Gdańsk, Av. Marszałka Piłsudskiego 46, 81-378 Gdynia, Poland

**Keywords:** Mercury, Biomagnification, Seabirds, Gray seals, Fishes, Hair

## Abstract

Mercury (Hg), aside from having high toxicity, is characterized by its ability to biomagnify in the marine trophic chain. This is an important problem especially in estuaries, or in the coastal zone, particularly near the mouths of large rivers. This study was conducted in the years 2001–2011, in the coastal zone of the Baltic Sea near to the mouth of the River Vistula, which is the second biggest river discharging into the Baltic. Mercury concentration was measured in the tissues and organs of cod, flounder, herring, seals (living in the wild and in captivity), great black-backed gulls, and African penguins from Gdańsk Zoo, and also in human hair. Penguins and seals at the seal sanctuary in Hel were fed only herring. In marine birds and mammals and in the pelagic herring, the highest Hg concentration was observed in the kidney and in the liver, while in cod and flounder (located on a higher trophic level) the muscles were the most contaminated with mercury. In gray seals living in the seal sanctuary, Hg concentration in all analyzed tissues and organs except the kidneys was lower in comparison with seals living in the wild. The comparatively small share of fish in the diet of local Polish people and their preference towards the consumption of herring contributed to low concentration of Hg in their hair. The protective mechanisms related to detoxification and elimination of mercury were shown to be more effective in the seals than in the penguins, despite the former consuming around 10 times more food per day.

## Introduction

Mercury (Hg) is one of the most toxic metals. It performs no known functions in living organisms and, in both humans and animals, demonstrates nephrotoxicity, immunotoxicity, neurotoxicity, and mutagenicity (O’Shea 1999). In the twentieth century, cases of mortality due to methylmercury poisoning following the consumption of mercury-contaminated fish were observed in Japan, Sweden, and Canada (Ebinghaus et al. 1999). The negative impact of Hg on human health has caused its concentrations to be monitored in many parts of the world, including the Baltic Sea (HELCOM), with studies tending to focus on the muscles, liver, and kidneys of fish, marine mammals, and birds. Higher concentrations of this metal are found in the livers of marine mammals than in terrestrial mammals and, in the case of seals, it is estimated that an excessive concentration of Hg close to 60 μg g^−1^ causes damage to that organ (AMAP 1998). High concentrations of Hg were determined in dead ringed seals (*Phoca hispida*, Schreber 1775) (Fant et al. 2001; Nymann et al. 2003), gray seals (*Halichoerus grypus*, Fabricius 1791) (Ciesielski et al. 2006), and harbor seals (*Phoca vitulina*, Linnaeus, 1758) (Ciesielski at al. 2006) in the course of several studies in the Baltic region. In the liver of a seabird, meanwhile, an Hg concentration >25 μg g^−1^ dw is considered to be of adverse effect (Kalisińska 2009). Twenty percent of cormorants (*Phalacrocorax carbo*) (Misztal Szkudlińska et al. 2013) and most of the dead herring gulls (*Larus argentatus*), both predatory species in the coastal zone of the southern Baltic Sea, demonstrated such a level in their liver. Although gulls are omnivorous birds, those with a more fish-based diet had a higher mercury load (Szumiło et al. 2013), and this indicates that fish is not only a source of nutritional value in the body of a predator but also of toxic substances.

Two major rivers deposit into the Polish coastal zone of the Baltic, the Vistula and the Oder, their basins constituting 12 and 8 % of the Baltic’s drainage area, respectively. As both of these rivers flow close to mining regions in their upper reaches (Silesia), the Baltic is therefore potentially exposed to mercury which has been transported from afar (HELCOM 2004). Due to the fact that Hg is methylated, accumulated, and biomagnified in each subsequent level of the trophic chain, especially in its most toxic form, methylmercury, there could be a real threat to predators at the top—in this case, mammals and seabirds which feed in the southern Baltic. The main goal of the present study, therefore, was to assess the exposure of these predators (seabirds and mammals) to mercury through food which is derived largely from the ecosystem of the southern Baltic. A focus was placed on commercial fish species which are frequently consumed in the area (herring, cod, and flounder) and on dead seals and gulls which were found along the coast of the southern Baltic. For comparison, research was also conducted on penguins from the zoo and gray seals from the seal sanctuary in Hel, both of which are exclusively fed on herring from the southern Baltic. Human hair was selected and assayed as an indicator of mercury exposure in our own species.

## Materials and methods

The studies were conducted in the Polish coastal zone of the Baltic Sea during the years 2001–2011. Fishes were caught by fishermen in the Gulf of Gdańsk and the open Baltic, while dead seals and seagulls were found along the Polish coast. Dead penguins from Gdańsk Zoo and marine mammals, gray seals, from the seal sanctuary in Hel, were also included in the research. Permission to collect and dissect dead gulls of the *Larus* genus was granted by the Regional Director of Environmental Protection in Gdańsk (RDOS-22-PN.II-6631-4-42/2010/ek) and the General Director for Environmental Protection (DOPozg1z-4200/II1169/2015/10/km). The results of the study have been partially presented at conferences—mostly in Polish (Bełdowska et al. 2009; Falkowska et al. 2010; Kosecka et al. 2012; Szumiło et al. 2013).

In the present study, total mercury (Hgtot) concentration results are presented for the following caught fish, dead seabirds, and dead marine mammals: cod (*Gadus morhua*) (*n* = 75; total length 28–109 cm), flounder (*Platichthys flesus*) (*n* = 47; total length 24–47 cm), and herring (*Clupea harengus*) (*n* = 75; total length 18–27 cm), collected 2006–2009; dead African penguins (*Spheniscus demersus*) (*n* = 4: three juveniles and one adult female) from Gdańsk Zoo, collected 2009–2011; dead great black-backed gulls (*Larus marinus*) (*n* = 5: four juveniles and one adult male) which had been wintering in the Gulf of Gdańsk, collected 2009–2010 (Szumiło et al. 2013); and dead seals (*Halichoerus grypus*) found along the Polish coast (*n* = 5 adults: 3 males and 2 females) and from the seal sanctuary (*n* = 4 adults: 2 males and 2 females) at the Hel Marine Station belonging to the Institute of Oceanography, University of Gdańsk, collected 2001–2007. It was not possible to identify the sex of the juveniles as they were not yet of reproductive age. A bird’s age was determined on the basis of its feathers (Olsen and Larsson, 2004). In great black-backed gulls, juvenile birds were those in their first winter plumage, adults in their fourth/final plumage. The penguins and seals from captivity had been fed exclusively on herring caught in the Polish waters of the southern Baltic. Samples of the herring were collected for Hg analysis once a month for 1 year. Given that both high and low concentrations of Hg were observed in the females and males and also taking into account previous studies, where there were no statistically significant differences in the Hg concentrations in males and females (Nyman et al. 2002; Szumiło-Pilarska et al. 2015), the sex was not taken into account.

Additionally, in the seal sanctuary, samples of excrement were collected six times during the year (*n* = 30). Samples of penguin guano were also collected at the zoo 12 times during the year (*n* = 94). Samples were collected from a teflon mat onto fiberglass filters.

These findings are complemented by those determined in human hair samples taken from 160 volunteers within the 20–25-year age group who, for most of the year, live in the Tricity (Gdynia-Sopot-Gdańsk) on the shores of the Gulf of Gdańsk. The hair was cut close to the skin then washed with acetone and deionized water. Eighty-three percent of the volunteers claimed to eat fish no more than three times a month.

For the different species collected, Hgtot concentrations were determined as follows: in fish, for the liver, spleen, heart, kidneys, intestines, muscle, brain, and gills; in seals, for the liver, kidneys, adrenal glands, muscles, lungs, heart, stomach, spleen, intestines, pancreas, and brain; and in birds, for the liver, kidneys, muscles, lungs, heart, and brain. The samples were obtained from dead animals through dissection. All samples of tissues and internal organs were stored at −20 °C. Prior to analysis, samples were freeze-dried and homogenized. Hg content was determined by use of an AMA 254 Advanced Mercury Analyzer (Leco^®^). Analysis used direct combustion in an oxygen-rich environment, the Hg being reduced to Hg(0) and subsequently transferred to its gas phase form, whereupon detection was conducted using conventional amalgamation-thermal desorption-AAS detection. This unique system easily analyzes samples in a short time and requires no pre-treatment or manual handling of the sample, thereby greatly reducing risk of contamination. Each sample was analyzed with a fivefold repetition. The analysis of certified reference materials (BRC-463 tuna fish) gave both satisfactory recovery and precision (RSD equal to 3 % of the mean). The detection limit for solid materials was set at 0.005 ng g^−1^ (Falkowska et al. 2013).

During each sample analysis, moisture content was measured so that the results could be presented both as wet weight and dry weight.

The results allowed for the calculation of the bioconcentration factor (BCF):

1 $$ \mathrm{B}\mathrm{C}\mathrm{F} = \mathrm{C}/{\mathrm{C}}_{\mathrm{WT}} $$

C—Hg concentration in the scale or the gill; C_WT_—Hg concentration in the seawater (2.5 ng dm^−3^, Saniewska 2013); the bioaccumulation factor (BAF):

2 $$ \mathrm{B}\mathrm{A}\mathrm{F} = \mathrm{C}/{\mathrm{C}}_{\mathrm{WT}} $$

C—Hg concentration in the particular tissue or organs; C_WT_—Hg concentration in the seawater (2.5 ng dm^−3^, Saniewska 2013); the biomagnification factor (BMF):

3 $$ \mathrm{B}\mathrm{M}\mathrm{F} = \mathrm{C}1\ /\ \mathrm{C}2 $$

C1—Hg concentration in the tissue or organs of predators; C2—Hg concentration in the analogous tissue or organs of herring.

## Results and discussion

### Fishes—food of birds and mammals

**Fig. 1:**
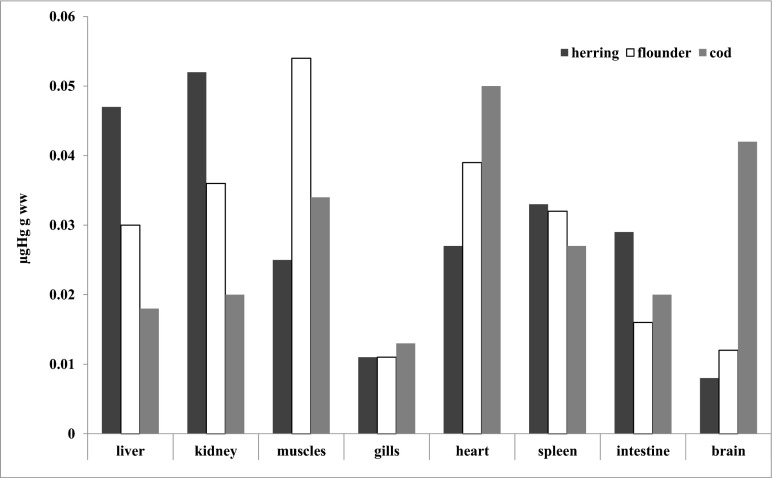
Medians of Hg concentration in organs of herring, flounder, and cod. Data have been presented as micrograms of Hg to grams of wet weight of samples

Total mercury concentrations in the muscle tissues of commercial fish collected in the Polish coastal zone of the southern Baltic have been observed to decrease on a multi-year scale (Polak-Juszczak 2009; Falkowska et al. 2010; Polak-Juszczak 2010). Median concentrations of Hgtot in the wet weight of muscles of fish caught 2006–2008 were slightly higher than the EU environmental target level (EQS) of 0.020 μg g^−1^ ww (Anonymous 2008) as follows: herring (median 0.025 μg g^−1^ ww), cod (median 0.034 μg g^−1^ ww), and flounder (median 0.054 μg g^−1^ ww) (Fig. 1). Only Hgtot concentrations measured in the muscles of sprat (median 0.014 μg g^−1^ ww) in 2003–2009 did not exceed the EQS value (Polak-Juszczak 2009).

Taking into account the various organs of fish, the lowest concentrations of Hg (below the EQS factor) were measured in gills, where inorganic mercury is adsorbed from the surrounding water. The gills are the most important path of entry for inorganic mercury into the body of a fish. Oliveira Ribeiro et al. (2002), based on experimental research involving Arctic char (*S. alpinus*), suggest a long-lasting effect of inorganic Hg on gill circulation. In organs where additional Hg is absorbed from food, low median concentrations (≤EQS value) were measured in the gonads, where chemical substances are removed during spawning, and in predatory cod in the organs responsible for elimination: liver 0.018 μg g^−1^ ww and kidneys 0.020 μg g^−1^ ww. In pelagic herring, the situation was reversed—the highest median Hgtot concentrations from the analyzed organs were for the liver 0.047 μg g^−1^ ww and kidneys 0.052 μg g^−1^ ww (Fig. 1). However, in the muscles, the average concentration of Hgtot was the lowest and equaled 0.025 μg g^−1^ ww. These differences were probably resultant upon the high lipid content of those muscles as the associated decrease in the amount of protein leads to reduction of mercury binding sites (Nakao et al. 2007). Hg concentrations were about two times higher in the muscles of predatory fish (cod 0.034 μg g^−1^ ww; flounder 0.054 μg g^−1^ ww) as a result of the fish being at a higher level in the food web and also the relatively low content of lipids (Kunachowicz et al. 2005).

The EU Water Framework Directive uses an additional parameter: background concentrations (BC) to the target value of 0.035 μg g^−1^ ww in fish muscles (Anonymous 2000). In this case, only total Hg concentration in the muscles of the benthic flounder exceeded this value (0.054 μg g^−1^ ww). However, in the muscles of sprat, herring, cod, and flounder caught in the Polish coastal zone of the Baltic Sea, the permitted level of Hg = 0.5 μg g^−1^ in fish muscle (WHO 1993) has not been exceeded (Polak-Juszczak 2009; Falkowska et al. 2010; Polak-Juszczak 2010). The highest concentrations of Hg were observed in the muscle (0.390 μg g^−1^ ww) and in the spleen (0.529 ng g^−1^ ww) of a single cod specimen.

In comparison to other regions, the fish caught in the Polish coastal zone of the Baltic Sea were not significantly contaminated with mercury. Furthermore, mercury concentrations in cod and herring caught off the Swedish coast were found to be at a similar level (HELCOM 2010; Bignert at al. 2011), indicating low regional variability of Hg levels in fish of the same species.

### Consumers of herring

Seals and seabirds are higher on the trophic chain of the Baltic than fish. In adult gray seals from the wild, total mercury concentrations in all analyzed tissues and organs, except for the kidneys, were several times higher (statistically significant) than in adult seals from the seal sanctuary (Table 1). This revealed significant ecological differences caused by diet. Seals from the seal sanctuary were fed only with herring which, in keeping with the findings of previous studies (Polak-Juszczak 2010; Falkowska et al. 2010; Falkowska et al. 2013), demonstrated lower Hgtot concentrations than other species of fish consumed by seals in the wild. Based on the stomach contents of nearly 300 wild gray seals, their diet was found to consist of the following: Atlantic herring (*Clupea harengus*)—85 % occurrence, European sprat (*Sprattus sprattus*)—30 % occurrence, common whitefish (*Coregonus lavaretus*)—17 % occurrence, sandeel (*Ammodytes spp*.)—12 % occurrence, viviparous blenny (*Zoarces viviparous*)—8 % occurrence, and Atlantic cod (*Gadus morhua*)—7 % occurrence. Cyprinids, Atlantic salmon, sea trout, flatfish, and perch also comprised a few percent each (Lundström et al. 2010). According to the authors, some differences may occur from year to year in the species composition of fish consumed by seals, with especially pronounced differences noted between the southern and the northern parts of the Baltic Sea. Assuming that each of the seals in the seal sanctuary consumes about 5 kg of herring daily, the average daily dose of mercury introduced into the organism is 46 μg. However, earlier studies point out that the daily dose of mercury depends on the age of consumed fish (Polak-Juszczak 2010; Falkowska et al. 2010).

**Table 1:** Mercury concentrations: median (minimum-maximum value) (μg g^−1^ dw) in tissues and organs of herring (*Clupea harengus*); flounder (*Platichthys flesus*); cod (*Gadus morhua)*; and seals (*Halichoerus grypus*) living in the Baltic Sea and in the sealarium, the penguin (*Spheniscus demersus)*, the great black-backed gull (*Larus marinus*), and the Great Cormorant (*Phalacrocorax carbo*)

Liver	Kidney	Adrenal	Muscles	Lung/gills	Heart	Stomach	Spleen	Intestine	Pancreas	Brain
*Clupea harengus* (Falkowska et al. 2010)										
0.14 (0.03–0.28)	0.19 (0.02–1.59)	No data	0.08 (0.03–0.26)	0.013 (0.001–0.04)	0.10 (0.03–0.036)	No data	0.12 (0.03–0.50)	0.12 (0.04–0.72)	No data	0.03 (0.01–0.11)
*Platichthys flesus* (Falkowska et al. 2010)										
0.06 (0.001–1.14)	0.13 (0.003–1.01)	No data	0.17 (0.003–0.79)	0.01 (0.004–0.08)	0.15 (0.001–1.56)	No data	0.12 (0.003–1.55)	0.07 (0.004–0.47)	No data	0.05 (0.004–0.44)
*Gadus morhua* (Falkowska et al. 2010)										
0.05 (0.01–1.12)	0.07 (0.01–0.48)	No data	0.11 (0.03–1.22)	0.02 (0.002–0.12)	0.19 (0.02–1.02)	No data	0.10 (0.03–1.96)	0.08 (0.01–1.22)	No data	0.18 (0.03–1.30
*Halichoerus grypus* in the wild										
297.88 (1.39–974.57)	12.05 (1.81–19.83)	4.17 (1.79–6.56)	1.81 (0.38–5.07)	2.36 (0.11–8.53)	1.18 (0.12–3.21)	1.84 (1.29–2.39)	45.32 (0.19–173.93)	0.98 (0.15–2.06)	1.63 (0.23-4.12)	0.33 (0.09–0.53)
*Halichoerus grypus* in the sealarium										
37.44 (1.22–57.96)	11.05 (0.71–16.05)	0.51 (0.48–0.54)	0.66 (0.39–0.82)	0.25 (0.17–0.43)	0.38 (0.16–0.59)	0.26 (0.21–0.31)	0.57 (0.30–0.85)	0.39 (0.37–0.41)	0.39 (0.24–0.54)	0.15 (0.14–0.17)
*Spheniscus demersus* (Falkowska et al. 2012)										
1.63 (1.46–3.04)	0.89 (0.63–1.82)	No data	1.13 (0.67–1.36)	0.87 (0.55–1.46)	0.90 (0.23–0.99)	No data	No data	No data	No data	0.56 (0.17–0.74)
*Larus marinus* (Szumiło et al. 2013)										
2.18 (0.61–6.81)	1.57 (0.92–4.44)	No data	0.53 (0.33–5.14)	0.88 (0.56–3.37)	0.58 (0,31–3.23)	No data	No data	No data	No data	0.51 (0.20- 1.57)
*Phalacrocorax carbo* (Misztal-Szkudlińska et al. 2013)										
7.71 (1.42–80.72	13.11 (1.25–298)	No data	1.83 (0.49–5.56)	2.43 (0.81–7.92)	No data	1.28 (0.31–4.67)	No data	1.39 (0.28–6.18)	No data	no data

Data of mercury concentration are presented on dry weight

The concentrations of Hgtot in the kidneys of seals in the wild and in captivity were comparable. This was probably due to faster elimination of the metal from kidneys than from liver.

The African penguin naturally inhabits the coast of southern Africa, but, in Gdańsk, it is bred in the zoo. The fact that these penguins are fed herring caught in the Gulf of Gdańsk makes them closely comparable to other marine predatory birds inhabiting the coastal zone of the southern Baltic (e.g., gulls, terns, guillemots, auks, and cormorants) and therefore a suitable control sample to use in research. In the penguins, as with the great black-backed gulls and the seals, the highest average Hgtot concentration among all the analyzed tissues was observed in the liver (1.69 μg g^−1^dw) (Table 1). Detoxification, transformation, and redistribution of contaminants all occur in the liver, and these processes have a significant impact on the health and condition of the animals. However, the level of metal concentration in the kidneys was similar to the values determined in the muscles, lungs, and heart (Table 1). This is the opposite to what was observed in seals from captivity, for which Hg concentrations in the liver were more than 20 times higher than those observed in penguin livers. This is mostly associated with the difference in food intake, the penguins consuming almost 10 times lower weight of fish. Taking into account the much lower concentrations of Hg in the muscles, heart, and brain of seals in captivity compared to the respective penguin tissues (Table 2), it can be suggested that the elimination of Hg in mammals is more effective than in penguins.

**Table 2:** The mean and minimum-maximum bioaccumulation factor (BAF) and the biomagnification factor (BMF) of Hg in tissues and organs of the penguin (*Spheniscus demersus*) from the zoo and the seal from the sealarium (*Halichoerus grypus*)

		Liver	Kidney	Lung	Muscles	Heart	Brain
*Spheniscus demersus*	BAF	64	33	29	34	22	16
		(46–95)	(20–57)	(17–46)	(21–43)	(7–31)	(5–23)
	BMF	13	5	–	10	7	8
		(9–19)	(3–8)	–	(7–14)	(2–10)	(3–11)
*Halichoerus grypus*	BAF	765	176	5	15	7	2
		(247–1206)	(12–275)	(3–7)	(8–21)	(3–12)	(2.7–3.0)
	BMF	182.5	24.4	–	7.7	3.2	5.4
		(6–288)	(2–38)	–	(4–11)	(1–5)	(5–6)

### Fish consumers

In the fish-eating great black-backed gulls, cormorants (Misztal-Szkudlińska et al. 2013), and seals, the highest median concentrations of Hg were observed in the liver and kidneys (Table 1). In the fish-eating *Larus marinus*, as in penguin and seals, the highest median Hg concentrations were measured in the liver (Szumiło et al. 2013), whereas in cormorants, Hg concentration in the liver was lower by half than in the kidneys (Misztal-Szkudlińska et al. 2011; Misztal-Szkudlińska et al. 2013). This is probably related to the type of food being caught in the coastal zone of the Gulf of Gdańsk and the Vistula Lagoon, which includes members of the *Cyprinidae* (carp) and *Percidae* (perch) families such as the following: ruffe (*Gymnocephalus cernuus*), perch (*Perca fluviatilis*), roach (*Rutilus rutilus*), herring (*Clupea harengus*), smelt (*Osmerus eperlanus*), and to a lesser degree tench (*Tinca tinca*) and Prussian carp (*Carassius gibelio*) (Stempniewicz and Grochowski 1997; Martyniak et al. 2003; Stempniewicz et al. 2003). Hgtot concentrations in the liver and kidneys were several times higher in the cormorants than in the penguins, fed only on herring, or in the great black-backed gulls. The diet of the latter consists largely of fish being hauled in by fishing boats, the viscera of fish gutted onboard these boats as they return to port, marine organisms found on the beach or in the shallow eulittoral area, and other municipal waste (Szumiło et al. 2013).

As a geochemical background value, mercury in the liver of birds has been defined as 3 μg Hg g^−1^ dw (Kalisińska 2009). Concentrations of this metal in the livers of great black-backed gulls and African penguins, for which the main food source had been fish from the Polish coastal zone of the Baltic, did not exceed this value. Hg concentrations were higher in the livers of cormorants, but most individuals did not exceed the value of 25 μg g^−1^ dw, above which there may be an adverse effect on the bird’s organism (Kalisińska 2009).

**Fig. 2:**
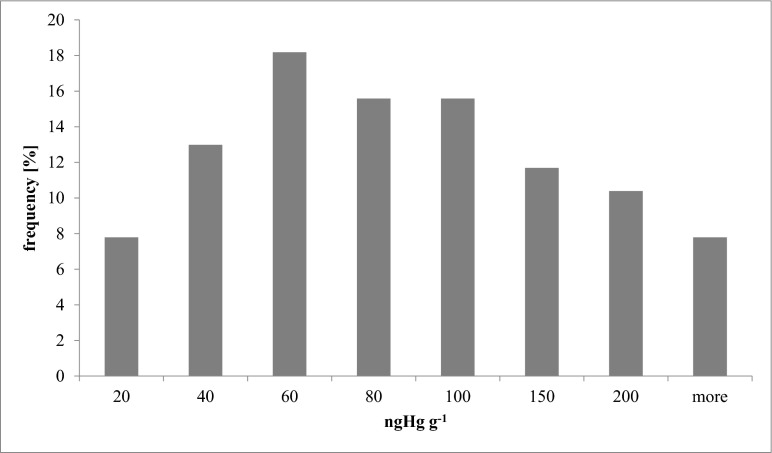
Histograms of Hg concentration in hair of Poles living in the Polish coastal zone of Baltic

Almost one million people live in the Tricity agglomeration (Gdańsk-Gdynia-Sopot) on the coast of the Gulf of Gdańsk, but, despite easy access to fresh products, the collective diet is not rich in fish and seafood. Among the adult volunteers who participated in the research, 87 % consumed fish no more than three times a month and the small contribution of fish to their diet was confirmed by the average Hgtot concentration in their hair (91.8 ng g^−1^) (Fig. 2). These results were much lower than in the countries with the highest fish and seafood consumption: Japan 1510 ng g^−1^ (Ohno et al. 2007) and Spain 638 ng g^−1^ (Díez et al. 2008). The majority of Hg concentrations measured in the hair of Poles fell within the range of 60–100 ng g^−1^ (Fig. 2). The lowest concentrations of Hg (<10 ng g^−1^) were measured in the 5 % of volunteers who declared that they did not eat any meat, fish, or seafood.

The low level of Hgtot concentrations in Poles was also confirmed in earlier studies by Lech and Sadlik (2004), where the levels of Hg in urine and liver were found to be an order of magnitude lower than those of Swedes, Norwegians, Spaniards, Danes, Japanese, and Koreans (Johansen et al. 2007; Julshman et al. 1989; Garcia et al. 2001; Suzuki et al. 1993; Yoo et al. 2002). However, high consumption of fish is not always reflected in high Hgtot levels in the hair of the consumer. Research conducted in the Okavango Delta in Botswana, where fish consumption is high among the local population, indicated that relatively low mercury concentrations in the hair of the inhabitants (210 ± 220 ng g^−1^) resulted from the small amount of mercury present in fish (19 ± 19 ng Hg g^−1^ ww in planktivorous fish; 59 ± 53 ng Hg g^−1^ ww in predatory fish) (Black et al. 2011). This observation may be made with regards to the lower Hg concentrations determined in the seals from the Hel sanctuary, fed only on herring, than in the wild seals feeding on various species of fish. The low mercury levels in the hair of Poles may result not only from low consumption of fish but also from the preferred species, as herring (which contains the lowest concentration of Hgtot among commercially caught fish in the region) is very popular (Falkowska et al. 2010).

### Bioconcentration, bioaccumulation, and biomagnification of Hg

Going up the trophic chain, the concentration and percentage of methylmercury in total mercury increase, reaching its highest values in the predatory organisms of seals and birds (Boening 2000; Houserová et al. 2007). Taking into account the average concentration of mercury (2.5 ng dm^−3^) in the Polish waters of the southern Baltic (Saniewska 2013), the bioconcentration factor BCF (1) was calculated for Hg in the scales and gills of cod, herring, and flounder. The concentration of Hg in the scales and gills of the fish was found to be more than 1000 times higher than in the surrounding water. As the respiratory system of fish has contact with the highest volume of water and is the most important route of entry for aqueous-phase inorganic mercury, the bioconcentration of Hg in the gills (BCF = 8 000) is therefore two times more efficient than on the scales (BCF = 4 000). In other tissues and organs, Hg absorption with food prevails.

The bioaccumulation factor BAF (2) for mercury in marine animals was calculated on the basis of the captive seals and penguins, for which the sole food source was whole herrings from the southern Baltic with average mercury concentration of 0.02 μg g^−1^ ww. Bioaccumulation calculations indicated an increase of Hg concentration in the tissues and the organs of the organisms as a result of prolonged exposure to the metal, mostly through food (Table 2). The particular ability of mercury in the environment to bioaccumulate in living organisms was expressed by the liver of a seal, both to the mean value and between individuals. Mercury BAF in the kidneys was four times lower and in the muscle 100-fold lower compared to the liver (Table 2). Based on Olsson’s theory (1976), the ratio for Hg concentration in the liver to Hg in the muscles is used to evaluate the accumulation of mercury in marine biota. Studies involving vertebrate animals, mainly fish, indicated that Hg_liver_/Hg_muscle_ > 1 reflects an increase in absorption over elimination, while an inverse relationship indicates a ratio of < 0.5 (Julshamn et al. 1982; Goldstein et al. 1996; Wiener et al. 2003; Amlund et al. 2007). Major differences between Hg concentrations in the livers and muscles of seals (Table 2) may therefore indicate increased absorption due to long-term dietary exposure. This results from the fact that mercury, mainly in the form of methylmercury, is transmitted to the circulatory system and in seals becomes accumulated relatively quickly in the liver, from where it is transferred to other tissues and organs.

In seal excrement, measured Hg concentrations were within the range of 0.1 to 0.8 μg Hg g^−1^ ww, whereas those in penguin guano were lower and ranged from 0.001 to 0.2 μg g^−1^ ww. This may indicate faster metabolism and more efficient elimination of mercury from the bodies of gray seals as compared to penguins. Consequently, in the muscles, heart, and brain—the final destinations for mercury within the organism—BAF values were several times higher in the penguins than in the seals. The ratio of fish intake weight to body weight was found to be comparable between the penguins and seals.

Taking into account the average concentration of mercury in the consumed whole herring (0.02 μg g^−1^ ww) and the mean concentrations of Hg in the feces of seals (0.1 μg g^−1^ ww) and penguin guano (0.04 ng g^−1^ ww), it was calculated that the Hg concentration was seven times higher in the feces compared with the food, and in the guano more than two times higher. Additionally, seals excrete further toxins with urine. Concentrations of Hgtot in the liver and kidneys of seals and birds showed the advantage of eliminating the accumulation, but it should be emphasized that a greater load of mercury was retained in the bodies of birds than seals.

As a result, the concentration of mercury in the penguin muscle was two times higher, and in the brain even three times higher, than in the seals, in spite of the greater daily food intake and higher daily dose of metal in the latter.

The BMF of Hg in the organs of the individual captive seals when compared to the same herring organs was at its highest in the liver (BMF = 183), while it was considerably lower in the kidneys (BMF = 24) and at its lowest in the muscle, heart, and brain (BMF from 8 to 3) (Table 2). In penguins, BMFs calculated for the liver and muscles were similar and amounted to 10–13, while the smallest biomagnification of mercury was recorded in the kidneys (BMF = 5). In terms of the liver and kidneys, the process of Hg biomagnification was higher in seals than in penguins. However, in the muscles, heart, and brain, an inverse trend was observed which showed that the same protective mechanisms (related to, e.g., demethylation, detoxification, and elimination of mercury) are more effective in seals than in penguins, despite the daily food consumption of seals being about 10 times greater (in mass) than that of penguins.

Taking into account the reports of Lundström et al. (2010), in which herring was the predominant dietary component for seals, it can be calculated that the concentration of mercury in the livers of dead seals found along the Polish coast was a thousand times higher, and in the kidneys dozens of times higher, than in the tissues of the fish caught in the Polish economic zone (Table 1). The mean concentration of Hg in the seals’ muscles was several times higher than in the muscles of herring. Although the level of Hg biomagnification in wild seals is unknown, their BMF values are probably at a similar level to those of captive seals.

## Summary

The fishes caught in the Polish coastal zone of the Baltic Sea were not more contaminated with mercury than the same species from other regions of the Baltic, and the obtained results demonstrate the lack of regional differences between fish of the same species when it comes to mercury levels. HELCOM reports indicate Poland as being a significant emitter of Hg into the Baltic Sea, but these are based to a large extent on modeling and extrapolation of data from other areas. Hg concentrations in fish caught in the southern Baltic and consumers of these fish—birds, mammals, and humans (hair)—do not confirm these reports. This indicates the requirement for more regular research in the Polish coastal zone, using modern equipment.

In herring, positioned on the lower level of the trophic web, the highest Hg concentrations occurred in the organs responsible for elimination and detoxification of the body: kidneys and liver. Mercury in the muscles, the target site of accumulation, was almost two times lower. In fish from a higher level of the food web (cod and flounder), accumulation in the muscle tissue prevailed. In marine birds and mammals, as with the herring, the highest Hg concentrations were observed in the kidneys and liver. Diet was observed to determine the level of Hg in marine organisms to a large extent. In adult gray seals living in the seal sanctuary and fed only on herring, mercury concentrations in all analyzed tissues and organs (except the kidneys) were lower in comparison with seals living in the wild. The herring caught in the Polish economic zone had the lowest concentration of mercury in comparison to other fish consumed by Baltic seals. The diverse diet of seals in the wild, on the other hand, led to Hg loads in viscera which were several times greater.

In terms of the local human population, the small contribution of fish to their diet and a preference towards consumption of herring contributed to low concentrations of Hg in their hair.

It is likely that the faster metabolism and more efficient elimination of mercury from the body demonstrated by gray seals, as compared to penguins, were responsible for Hg concentrations in the penguins’ muscles and brains being twice and as much as three times higher, respectively, than in the seals. This demonstrates that the protective mechanisms related to the demethylation, detoxification, and elimination of mercury were more effective in the seals than the penguins, despite the former consuming around 10 times more food mass per day and therefore having a far greater daily intake of mercury.
